# Electrical Characteristics and pH Response of a Parylene-H Sensing Membrane in a Si-Nanonet Ion-Sensitive Field-Effect Transistor

**DOI:** 10.3390/s18113892

**Published:** 2018-11-12

**Authors:** Bo Jin, Ga-Yeon Lee, ChanOh Park, Donghoon Kim, Wonyeong Choi, Jae-Woo Yoo, Jae-Chul Pyun, Jeong-Soo Lee

**Affiliations:** 1 Department of Electrical Engineering, Pohang University of Science and Technology, Pohang 37673, Korea; shengzhi86@postech.ac.kr (B.J.); kdong620@postech.ac.kr (D.K.); pathfinder@postech.ac.kr (W.C.); 2 Department of Materials Science and Engineering, Yonsei University, Seoul 03722, Korea; gayeon@yonsei.ac.kr; 3 Division of IT Convergence Engineering, Pohang University of Science and Technology, Pohang 37673, Korea; chduckling@postech.ac.kr; 4 IM Healthcare Co. Ltd., Yongin 26354, Korea; jwyoo@im-healthcare.com

**Keywords:** parylene-H, ion-sensitive field-effect transistor (ISFET), pH response

## Abstract

We report the electrical characteristics and pH responses of a Si-nanonet ion-sensitive field-effect transistor with ultra-thin parylene-H as a gate sensing membrane. The fabricated device shows excellent DC characteristics: a low subthreshold swing of 85 mV/dec, a high current on/off ratio of ~10^7^ and a low gate leakage current of ~10^−10^ A. The low interface trap density of 1.04 × 10^12^ cm^−2^ and high field-effect mobility of 510 cm^2^V^−1^s^−1^ were obtained. The pH responses of the devices were evaluated in various pH buffer solutions. A high pH sensitivity of 48.1 ± 0.5 mV/pH with a device-to-device variation of ~6.1% was achieved. From the low-frequency noise characterization, the signal-to-noise ratio was extracted as high as ~3400 A/A with the lowest noise equivalent pH value of ~0.002 pH. These excellent intrinsic electrical and pH sensing performances suggest that parylene-H can be promising as a sensing membrane in an ISFET-based biosensor platform.

## 1. Introduction

The ion-sensitive field-effect transistor (ISFET) sensor is a potential candidate for future bioassay applications due to its low cost, fast response, high sensitivity and small sensing size. Recently, studies have been conducted on channel materials such as carbon nanotubes and graphene-based materials [1,2]; channel structures such as silicon nanowire (Si NW) arrays, Si-nanonet structure and suspended Si NW [3,4,5]. Other approaches to improve the sensing responses have been made by introducing alternative sensing materials instead of SiO_2_ as the gate insulator in ISFET. Several issues related to sensing membranes such as insufficient isolation between the electrolyte and ISFET channel and dangling bonds in the sensing membrane can degrade the sensing responses, reliability and lifetime [6,7].

Parylene (polymer of *p*-xylene), which is extensively used as a biocompatible encapsulant for implantable microdevices [8], can be utilized as an ISFET gate insulator due to its high electrical resistivity. More recently, a new parylene modified with a formyl group (parylene-H) was proposed as a sensing membrane in microplate-based immunoassay and surface plasmon resonance (SPR) biosensor applications [9,10]. It has been demonstrated that the formyl group of parylene-H can covalently bond to the primary amine group of target molecules without any additional surface modification steps [10].

In this paper, we fabricated Si-nanonet ISFETs with parylene-H gate insulator (p-H ISFETs) and investigated their DC and reliability characteristics. We also evaluated the pH sensitivity of the p-H ISFETs in various buffer solutions and conducted low frequency noise analysis for potential development in bioassay applications.

## 2. Experimental Section

### 2.1. Device Fabrication

The p-H ISFETs were fabricated on an 8-inch silicon on an insulator substrate comprising a 100-nm top Si layer (<100> oriented, boron-doped, 10 Ω·cm) and a 400-nm buried oxide layer. The active region was formed by photolithography and inductively-coupled plasma reactive etching (ICP-RIE). To form the source and drain regions, arsenic ions with a dose of 5 × 10^15^ cm^−2^ were implanted by an ion implantation process with masking the ISFET active channel region by photoresist, and post-annealing at 1000 °C for 20 s was performed to activate the dopants. Then, the nanonet structure was defined on the ISFET channel region by electron-beam lithography and ICP-RIE. A metal layer of ~200 nm was deposited and patterned by a lift-off process on the source and drain regions to provide low contact resistance. A 1.5-μm SU-8 layer was coated to provide a passivation layer while opening the FET channel and the source and drain contact pad regions.

The parylene-H layer was thermally deposited by the following polymerization steps: (1) evaporation of parylene dimers at 160 °C, (2) production of a highly reactive *p*-xylene radical by pyrolysis at 650 °C and (3) deposition of the wafer at room temperature [11,12]. To control the thickness of the parylene-H layer, the quartz crystal microbalance response was measured from the beginning of the evaporation step. Finally, after the deposition of the parylene-H layer so as to form the gate insulator, the contact pad regions were opened.

### 2.2. Apparatus

The entire parylene-H coating procedure was reproducibly conducted using a microprocessor-controlled parylene coater. The morphology of the as-fabricated ISFET channel was observed by scanning electron microscopy (SEM), and the thickness of parylene-H was confirmed by atomic force microscopy (AFM). The electrical characteristics of the fabricated ISFETs were measured for current-voltage (I-V) with a Keithley 4200-SCS analyzer and capacitance-voltage (C-V) with an Agilent 4284A Precision LCR Meter at room temperature. Low frequency noise characteristics were measured with a noise analyzer Cadence BTA9812A/B and a vector signal analyzer Agilent 89441A. The gate bias was applied to the liquid gate through the external Ag/AgCl reference electrode. All the electrical characterizations of the devices were performed in 0.01× PBS solution at room temperature.

**Figure 1:**
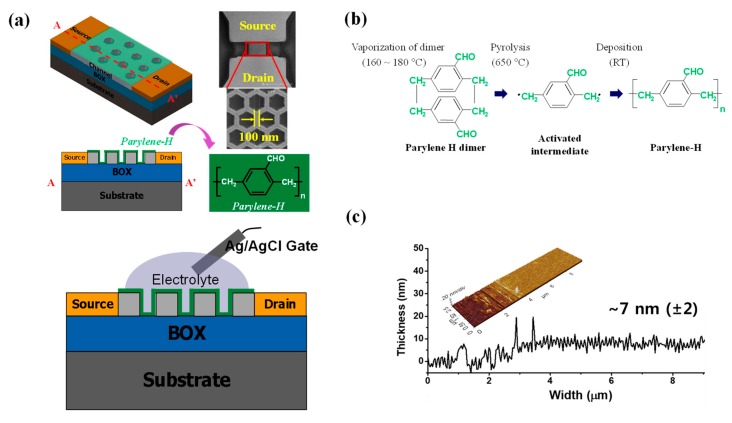
(**a**) Schematics and top-view SEM images of the fabricated Si-nanonet ISFET with parylene-H as the gate insulator. (**b**) Illustration of the parylene-H deposition process. (**c**) AFM images of the parylene-H nanolayer with a 7-nm thickness.

Si-nanonet ISFETs with a 100-nm branch width were fabricated, as shown in Figure 1a. The illustration of the parylene-H deposition process is shown in Figure 1b. A 7 nm-thick parylene-H layer was deposited on the nanonet channel. The thickness was measured using AFM, as shown in Figure 1c.

## 3. Results and Discussion

### 3.1. DC and Reliability Characteristics of p-H ISFETs

**Figure 2:**
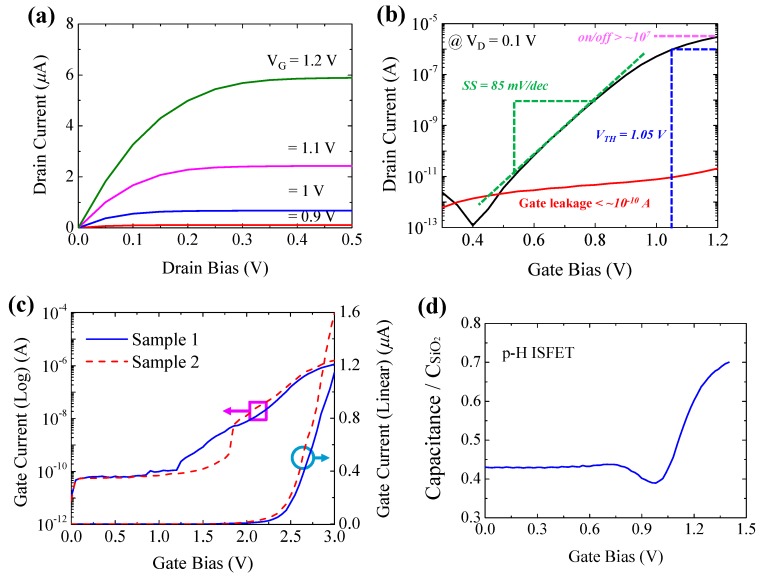
Electrical characteristics of the fabricated parylene-H (p-H) ISFET in 0.01× PBS buffer solution. (**a**) Typical I_D_-V_D_ output characteristics in the linear scale. (**b**) Typical I_D_-V_G_ transfer characteristics and I_G_-V_G_ gate leakage current characteristics at V_D_ = 0.1 V in log scale indicating a typical n-type behavior. (**c**) I_G_-V_G_ characteristics in log and linear scale. (**d**) Normalized C-V characteristics of the p-H ISFET.

To investigate the DC characteristics of parylene-H as a gate insulator, the output characteristic curve (I_D_-V_D_) and transfer curve (I_D_-V_G_) of the p-H ISFET with a 7 nm-thick parylene-H were measured, as shown in Figure 2. The drain bias (V_D_) was swept from 0–0.5 V by applying the liquid gate bias (V_G_) from 0.9–1.2 V in steps of 0.1 V. The drain current (I_D_) showed a linearly increasing behavior as explained by I_D_ ∝ (V_G_-V_TH_)·V_D_ at low V_D_, while the saturation condition was obtained at higher V_D_ as explained by I_D_ ∝ (V_G_-V_TH_)^2^, indicating typical n-type FET behaviors. In Figure 2b, V_G_ was swept from 0.3–1.2 V at constant V_D_ = 0.1 V. Excellent electrical characteristics were obtained with a high I_D_ on/off ratio of >10^7^, a low subthreshold swing (*SS*) of ~85 mV/dec, a low threshold voltage (V_TH_) of 1.05 V at a constant current level of 10^−6^ A and a low gate leakage current of <10^−10^ A in the whole swept range. These DC characteristics suggest that parylene-H shows outstanding electrical properties in electron device applications, replacing conventional silicon dioxide material.

To further evaluate the reliability of parylene-H as a gate insulator, the breakdown behavior was investigated by applying gate bias stress from 0–3 V. As shown in Figure 2c, the gate current (I_G_) increased with the applied gate bias. Within the range of V_G_ = 1.5–2.5 V, the I_G_ showed a soft breakdown where an abnormal increase of the leakage current at relatively lower gate bias was observed; over V_G_ = 2.5 V, the I_G_ increased greatly, leading to a hard breakdown [13]. By C-V characterization, both of the capacitances of p-H ISFET and SiO_2_ gate insulator ISFET (Ox ISFET) with the same channel area were measured, and Figure 2d shows the normalized C-V characteristics of the p-H ISFET. The capacitance of p-H ISFET was extracted to be 3.92 × 10^−7^ F/cm^−2^, and therefore, the dielectric constant of parylene-H was estimated to be ~3.1, comparable to the 2.6–3.2 range of the parylene series as reported in the literature [14]. Therefore, the equivalent oxide thickness (EOT) of a 7 nm-thick parylene-H in p-H ISFET was calculated as low as ~8.8 nm.

The interface trap density (*N_it_*) at the Si/parylene-H interface was extracted by the following equation [15]:

(1) $$N_{it}=\left[\left(\frac{SS}{\ln10}\right)\left(\frac{q}{\mathrm{kT}}\right)-1\right]\cdot \frac{C_{i}}{q},$$

where *q* is the electron charge, k is the Boltzmann constant, T is the absolute temperature and *C_i_* is the capacitance of the gate insulator per unit area. The *N_it_* of p-H ISFET was estimated to be as low as ~1.04 × 10^12^ cm^−2^. The field-effect mobility (*μ*_FE_) in the linear region was also extracted based on the following equation [15]:

(2) $$\mu_{\mathrm{FE}}=\frac{L\cdot g_{m.\max}}{W\cdot C_{i}\cdot V_{D}},$$

where *L* is the channel length, *W* is the channel width and *g_m_*_.max_ is the maximum transconductance. The extracted *μ*_FE_ of our p-H ISFETs reached as high as ~510 cm^2^V^−1^s^−1^.

### 3.2. Sensitivity of p-H ISFETs

**Figure 3:**
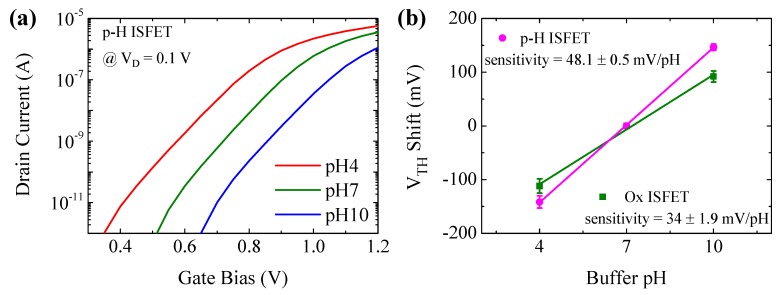
pH-dependent filed-effect characteristics. (**a**) I_D_-V_G_ transfer characteristics at V_D_ = 0.1 V in pH 4, 7, 10 buffer solutions for a p-H ISFET. (**b**) V_TH_ shift indicating a linear pH response: the sensitivity of 48.1 ± 0.5 mV/pH and device-to-device variation of ~6.1% for p-H ISFETs; the sensitivity of 34 ± 1.9 mV/pH for Ox ISFETs.

To evaluate the pH sensor response of the p-H ISFETs for potential bioassay applications, the output characteristics were measured at pH = 4, 7 and 10 buffer solutions by sweeping V_G_ from 0.3–1.2 V at constant V_D_ = 0.1 V. Figure 3a shows the I_D_-V_G_ curve shifts as a function of pH value in the buffer solution. Figure 3b shows the change of V_TH_ of the p-H ISFETs measured in different pH solutions. The calculated pH sensitivity (S_pH_) was as high as 48.1 ± 0.5 mV/pH, which is ~40% higher than that of ISFETs with SiO_2_ gate insulator (Ox ISFET). The device-to-device variation was as low as ~6.1%. The pH sensitivity of ISFETs with a bare SiO_2_ membrane was reported to be 34 mV/pH and can increase up to 45 mV/pH with surface modification by functional molecules [16]. The parylene-H layer deposition process can simply control the formyl group concentration without additional surface modification. Our p-H ISFETs without any surface modification was comparable or even superior to other Si NW-based sensor platforms (see Table 1) reported in the literature [3,16,17,18]. The high sensitivity of p-H ISFETs is attributed to the formation of a high density formyl group (H–C=O) at the surface of the parylene-H/electrolyte, which can release or capture H^+^ [9,19]. Formyl groups can have polarity due to the difference in electro negativity, and protons can attach to carbon by electrostatic interaction. The p-H ISFETs have higher pH sensitivity than Ox ISFETs due to the higher density of hydrogen ions at the gate insulator/electrolyte interface, which contribute to inducing carrier generation in the ISFET channel.

### 3.3. Noise Analysis of p-H ISFETs

**Figure 4:**
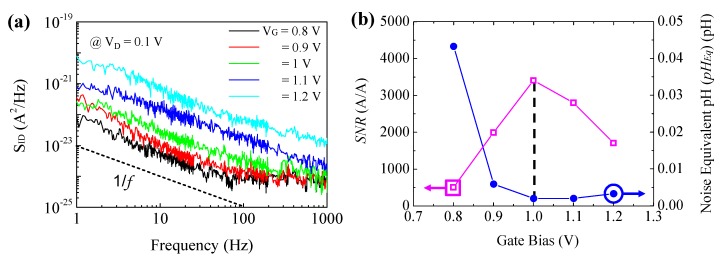
(**a**) Low frequency drain current noise characteristics of the p-H ISFET at various gate biases at V_D_ = 0.1 V. (**b**) SNR and noise equivalent pH vs. gate bias.

Noise is another important characteristic because it can fundamentally limit the sensitivity and resolution of the p-H ISFETs. The low frequency noise measurements of our p-H ISFETs were performed in pH = 7 buffer solution under various gate bias conditions. Figure 4a shows the drain current noise spectra S_ID_ vs. frequency at V_G_ = 0.8–1.2 V in steps of 0.1 V with constant V_D_ = 0.1 V. The S_ID_ shows a typical 1/*f*^α^ behavior with the exponential slope α ~ 1 in a 3-dec frequency bandwidth of ƒ_1_~ƒ_2_ = 1–1000 Hz. The signal to noise ratio (SNR) of our p-H ISFET was extracted based on the following equation [20]:

(3) $$SNR=\Delta I/\sqrt{\overset{{ƒ}_{2}}{\underset{{ƒ}_{1}}{\int }}S_{\mathrm{ID}}\left(ƒ\right)dƒ,}$$

where Δ*I* is the drain current change in the range of pH = 4–10. The noise equivalent pH (pH_Eq_) of our p-H ISFETs as determined by *SNR* and sensitivity was evaluated using the following equation [21] with *SNR* = 1,

(4) $${\mathrm{pH}}_{\mathrm{Eq}}=\sqrt{\overset{{ƒ}_{1}}{\underset{{ƒ}_{2}}{\int }}S_{\mathrm{ID}}\left(ƒ\right)dƒ}/\left(g_{m}S_{\mathrm{pH}}\right),$$

where *g_m_* is the transconductance. The extracted *SNR* and pH_Eq_ are shown as a function of gate bias in Figure 4b. The highest *SNR* of ~3400 A/A and the lowest pH_Eq_ of ~0.002 pH were obtained at V_G_ = 1 V for our p-H ISFETs, clearly indicating that the p-H ISFETs should be operated at near threshold voltage.

## 4. Conclusions

In conclusion, we proposed a p-H ISFET with a parylene-H sensing nanolayer. The excellent electrical properties of our p-H ISFETs such as subthreshold swing, threshold voltage, drain current on/off ratio and gate leakage current have been characterized. The low Si/parylene-H interface trap density and high field-effect mobility demonstrated the excellent electrical performance of our devices. In addition, it was demonstrated that the highly sensitive pH-dependent behaviors of p-H ISFETs exhibited pH sensitivity as high as 48.1 ± 0.5 mV/pH, which is ~40% higher than that of the conventional SiO_2_ gate insulator-based ISFETs. Furthermore, in accordance with the low frequency noise analysis, the SNR and noise equivalent pH of p-H ISFETs were investigated for potential development in ISFET-based biosensor applications. Future work will address p-H ISFETs for use in various biomarker sensing.

## Figures and Tables

**Table 1:** Comparison of the characteristics of Si nanostructure-based ISFETs.

Gate Insulator and Sensing Membrane	Device Channel	*SS* (mV/dec)	On/Off Ratio	Surface Treatment	pH Sensitivity (mV/pH)	Ref.
parylene-H	Si Nanonet	~85	>10^7^	w/o ^1^	48.1 ± 0.5	This work
SiO_2_	Si Nanonet	~63	>10^7^	w/o	35	[5]
SiO_2_	Si NW	/	/	w/o	34 ± 2	[16]
SiO_2_	Si NW	/	/	w ^2^	45 ± 0.3	[16]
SiO_2_	Si NW	~600	>10^5^	w	48 ± 1	[17]
SiO_2_	Si NW	~150	>10^5^	w	43 ± 3	[3]
Ta_2_O_5_	Si NW	~300	>10^3^	w	51.8 ± 0.1	[18]

^1^ w/o—without; ^2^ w—with.
